# Proinflammatory factors inhibition and fish oil treatment: A promising therapy for neonatal seizures

**DOI:** 10.1016/j.ibneur.2024.09.006

**Published:** 2024-10-02

**Authors:** Zohreh Ghotbeddin, Nima Badripour, Hossein Amini-Khoei, Zahra Basir, Shima Balali-dehkordi

**Affiliations:** a Department of Basic Sciences, Faculty of Veterinary Medicine, Shahid Chamran University of Ahvaz, Ahvaz, Iran; b Stem Cell and Transgenic Technology Research Center, Shahid Chamran University of Ahvaz, Ahvaz, Iran; c Medical Plants Research Center, Basic Health Sciences Institute, Shahrekord University of Medical Sciences, Shahrekord, Iran; d Department of Histology, Faculty of Veterinary Medicine, Shahid Chamran University of Ahvaz, Ahvaz, Iran; e Department of Basic Sciences, Faculty of Veterinary Medicine, Shahrekord University, Shahrekord, Iran

**Keywords:** Hypoxia, Fish oil, Behavioral performance, Pro-inflammatory factors, Histological changes, Rat

## Abstract

Brain injury is one of the most important causes of infant mortality and chronic neurological disabilities. Hypoxia is an acute brain injury which led to various cognitive, behavioral, and memory disorders throughout life. Previous studies reported neuroprotective possibilities for fish oil (FO) in brain-injured situations. In this study, we evaluated the effect of the FO diet during the lactation period on seizure activity, behavioral performance, histomorphometry, and inflammatory changes in the brains of hypoxia rats. Male Wistar rats were randomly divided in to 4 groups: Sham (intact rats), hypoxia, FO and FO+hypoxia groups. Hypoxia was induced by keeping neonate rats at PND12 in a hypoxic chamber (7 % oxygen and 93 % nitrogen intensity) for 15 minutes. In the FO groups, rats received oral FO (1 ml/day) for 12 days during the lactation period. Seizure activity was assessed by measuring the number of tonic-clonic seizures and seizure thresholds. Novel object recognition tests (NORT), rotarod, and open field tests were used to measure behavioral performances. A Histological study was performed to evaluate histomorphometric changes in the hippocampus and cerebellum. The gene expression of tumor necrosis factor-α (TNF-α) and interleukin-1β (IL-1β) was measured using RT-PCR**.** Findings showed that the number of tonic-clonic seizures, atrophy, and cell death in the hippocampus and cerebellum, the gene expression of TNF-α and IL-1β in the hippocampus, and behavioral disorders were significantly increased in the hypoxia rats compared to the sham group. Administration of FO in the hypoxia groups significantly decreased the gene expression of TNF-α and IL-1β, the number of tonic-clonic seizures, and neuronal cell death in the hippocampus and cerebellum compared to the hypoxia groups. Furthermore, it can improve behavioral tasks and cognitions.

## 1 Introduction

About 1–3 babies born in every 1000 births have a seizure in infancy. The neonatal period is the most vulnerable time for the occurrence of seizures. Neonatal seizures are often considered a clinical challenge due to the frequency of behavioral disorders in the long term. The immature brain is highly sensitive to oxygen reduction due to the strong dependence of neurons on the level of oxygen. Lack of access to oxygen for a few minutes can induce cell death of neurons (Vegda et al., 2022, Hamdy et al., 2020).

Cerebral hypoxia, which is defined as a relative lack of oxygen, is the most common cause of seizures in infancy. Hypoxia can cause many neurological disorders such as hyperactivity, learning disorders, lack of mental development, and behavioral disorders. It can also cause spontaneous and tonic-colonic seizures (Zhou et al., 2021, Koh et al., 2004, Roumes et al., 2022).

From a cellular and molecular view, hypoxia causes complex pathological changes that may lead to tissue necrosis through an inflammatory response. In general, hypoxia causes a lack of energy, increased secretion of excitatory amino acids such as glutamate, abundantly entry of sodium and calcium into cells, microvascular damage, apoptosis, inflammation, oxidative stress,and necrosis (Roumes et al., 2022).

Many long-term responses to hypoxia are mediated by Hypoxia-inducible-factor-1 which is an important regulator under hypoxia conditions. Hypoxia leads to an increase in the expression of inflammatory cytokines. It also activates microglia and astrocytes. TNF-α is one of the most important inflammatory cytokines that participates in the inflammatory responses in neurodegenerative diseases. However, the role of TNF-α during hypoxia is not fully understood (Eltzschig et al., 2014, Subedi et al., 2020, Yang et al., 2022, Zhang et al., 2013).

Additionally, hypoxia causes the over-expression of the C2OX an enzyme in microglia cells leading to the occurrence of inflammatory reactions in neurons (Farrell et al., 2016, Shiow et al., 2017).

Brain tissue is highly sensitive to lack of oxygen, and the reduction of oxygen level changes the function of neurons and ultimately causes neural death (Marutani et al., 2021). Spatial memory and learning disorders are caused by increased oxidative stress in areas of the brain that are involved in memory and learning, such as the hippocampus (Abdel-Wahab and Abdel-Wahab, 2016).

Hypoxia leads an increase in anaerobic glycolysis in the cells, increased oxidative phosphorylation, inflammation, and neuronal damage. Hypoxia also induces neurodegeneration and apoptosis. Systemic inflammation is the main mechanism of tissue damage following hypoxia. In general, chronic systemic inflammation following hypoxia causes tissue damage and behavioral disorders through the following ways: prolonged activation of microglia which causes neuronal damage (Ji et al., 2021, Wang et al., 2022), morphological changes in dendritic spines of neurons and downregulation of genes related to growth factors that are involved in learning and memory (Tu et al., 2023). Neural plasticity refers to the ability to change the strength of connections within the nervous system through injury or experience. Although it is one of the main features of the neuronal system in regulating learning, mood, and behavior, Hypoxia can disturb it (Appelbaum et al., 2023).

Docosahexaenoic acid (DHA) is one of the long-chain omega-3 unsaturated fatty acids. Fish oil (FO) is the main source of these fatty acids. DHA is found in the phospholipid membrane of cells, especially in neurons. DHA is an essential substance for the growth and development of the brain during the fetal and neonatal periods. The important benefits of DHA are in the transmission of nerve messages, the protection of nerve tissues, neurogenesis, and the protection of the brain from inﬂammation and injury by various mechanisms (Saini and Keum, 2018). It seems a diet rich in FO can affect the brain in morphology, especially in areas that play key roles in memory, behavioral,and executive functions (Hopperton et al., 2016).

Considering what was reviewed in general, the purpose of this research is to study the effect of FO treatment during breastfeeding on convulsive activity, behaviors, memory, histomorphometric changes, and gene expression of pro-inflammatory cytokines in the brain of hypoxic male rats.

## 2 Materials and methods

### 2.1 Animal

To perform this research, 12-day-old Wistar rats with an approximate weight of 18–22 g were used (7 rats in each group). Rats were kept in a completely standard laboratory condition (daily exposure of 12 hours of light to 12 hours of darkness, at a temperature of 22 ± 2 C and a humidity of 55 ± 5 %, and in independent cages with free access to water and food). All procedures were designed and executed in accordance with the protocols and guidelines. The current study was approved by the Institutional Ethics Committee of the Shahid Chamran University of Ahvaz (EE/98.24.3.26545/scu.ac.ir) and was conducted to the ARRIVE guidelines. To prevent the effect of the animal's circadian rhythm on the experiments, all the experiments were performed during day light hours, between 8:00 am and 4:00 pm. The animals were transferred to the laboratory one hour before the test to get adapted to the conditions of the laboratory.

### 2.2 Study design

Pregnant rats were randomly divided into 4 groups: the control group received saline (sham), the group received fish oil, the hypoxia group received saline, and hypoxia group received fish oil. No special treatment was done in the control group. The hypoxia groups were placed in the hypoxia box for 15 minutes with an intensity of 7 % oxygen and 93 % nitrogen (Sedláčková et al., 2014, Nalivaevaa et al., 2004). In the control and fish oil groups, hypoxia was not applied. Before being placed in the hypoxia box, the mothers of the rats in the control and hypoxia groups were given 1 ml of saline for 12 days during lactation period, and the mothers of the other groups received fish oil (1 ml from Sigma-Aldrich with a density of 0.93 g/ml) was used for 12 days at the lactation time (Kandratavicius et al., 2014, Aliheydari et al., 2020). A hypoxia box was used to apply hypoxia and induce convulsions. This box is a glass container and contains a fan, and an air inlet an outlet valve that is connected to an oxygen capsule and a nitrogen capsule. 12-day-old pups were placed inside the box and hypoxia was applied for 15 minutes by introducing 7 % oxygen and 93 % nitrogen into the box and adjusting this intensity with the help of an oximeter (Sedláčková et al., 2014, Nalivaevaa et al., 2004).

### 2.3 Evaluation of seizures activity

On the 12th day of birth and during a period of 15 minutes hypoxia induction, the number of tonic-clonic seizures was counted and the duration of the seizure threshold (the time interval between the application of hypoxia and the onset of the first seizure) was recorded by a stopwatch.

### 2.4 Behavioural evaluations

Behavioral tests including Novel Object Recognition Test, Open field, Rotarod, and Wire hanging test, were performed from PND43 to PND49. The Novel Object Recognition Test was used to assay cognition. Motor activity evaluated by Open field. Motor coordination and balance were measured respectively by Rotarod and Wire hanging tests.

#### 2.4.1 Novel Object Recognition Test (NORT)

To perform the NORT test, an open box-like playroom with walls measuring 70 ×70 cm and 50 cm high was used. A camera on the top of the chamber and a video recorder were used to monitor and record the animal's behavior. This method was carried out during three stages of habituation, training, and testing in a quiet environment with a constant amount of light. In the habituation phase, each rat was first placed in an empty chamber without objects in the operant space and was allowed to freely explore the area inside the chamber for 10 minutes. In the training phase, which took place 24 hours after the first phase, two identical objects (cubes) were placed in the chamber at a distance of 5 cm from the wall and the rat was allowed to explore the objects for 10 minutes. On the test day, 24 hours after familiarization with two identical objects, one of the objects was replaced by a new object, and the objects were placed in the same places, then each rat was placed in the chamber for 10 minutes. Exploratory behavior was defined as pointing the nose to the object at a distance of 2 cm or touching it with the nose or forehead. Each period was recorded on video and subsequently the duration of exploration for the old and new object was recorded manually with a stopwatch. In between the experiments, objects and the chamber were cleaned with 70 % ethanol to prevent odors. The indicators of the average discrimination ratio (DR) (the ratio of the search time for the new object to the total search time for both objects) and the difference score (DS) ( the difference between the time of new and old objects) were used for evaluations (Bevins and Besheer, 2006).

$DR=\frac{T_{\left(new\right)}}{T_{\left(total\right)}}$

$DS=T_{\left(new\right)}-T_{old}$

#### 2.4.2 Open field test

In this test, the movement activity of the animal, based on moving from one point to another, is measured and calculated by a camera. This device consists of an open cube box with a wooden floor and black walls measuring 50 ×50 x 50 cm. After setting the device and cleaning the inner wall of the cubic box, the animal is placed in the center of the box, and the first 5 minutes are considered for the animal's adaptation to the device. Then the total distance traveled, body stretching or standing on two legs and self-treatment for 5 minutes were recorded (Ghotbeddin et al., 2020).

#### 2.4.3 Rotarod

The rotarod device was used to check the balance activities and the power of motor coordination between the limbs. To evaluate the balance activity by this device, the animal was placed on a rotating horizontal bar with a diameter of 5 cm, speed was increased from 5 to 45 rpm in 300 seconds, and the time to maintain balance was recorded. First, each animal was given two opportunities to get used to and adapt to the device, and then the animal was placed on the device three more times with an interval of 2 minutes, and the average time obtained was calculated. It should be noted that the duration of the rat's stay on the rod was determined to be a maximum of 300 seconds, and the habituation phase and the test were performed in one day (Monville et al., 2006).

#### 2.4.4 Wire hanging test

A wire hanging test was used to measure the strength and balance of the rats. 40 cm diameter circular lace with 1 cm diameter lace squares. The rat was placed in the middle of this net and immediately the net was reversed and kept at a distance of 50 cm above the ground. The maximum test time was 120 seconds. The method of scoring and evaluating the wire hanging test was based on how long the rat stayed on the net. The mean of these times (seconds) was recorded. The experiment was conducted in triplicate (Mohammad Ali Mansouri et al., 2018).

### 2.5 Sampling

After the behavioral tests at PND 49, all the rats in the experimental groups were anesthetized by ketamine (100 mg/kg and xylazine (10 mg/kg),and the hippocampus was quickly removed and placed in a 10 % buffered formalin solution and or immediately frozen in liquid nitrogen and keep in freezer −70 ℃ until assessments.

#### 2.5.1 Histological assessments

For histological studies, the isolated brain was placed in formalin, and after going through the usual histological procedures, 5-micron sections were cut from it and finally stained with hematoxylin and eosin (Merck, Germany) and studied under a light microscope. Tissue passage includes the steps of sampling and proofing, washing the fixative with running water, dewatering, clarifying, impregnating with paraffin, molding, cutting, staining, and attaching the slide (Slaoui and Fiette, 2011). The measurement of Purkinje cells height was done using the Dino-Eye lens (AM7023, Taiwan), and Capture 2.0 computer software.

### 2.6 Real-time PCR analysis for gene expression

The mRNA expression level of TNF-α and IL-1β was evaluated using real-time PCR in the hippocampus. At first, total RNA was isolated from the frozen hippocampus samples using the TRIzol reagent (Invitrogen). RT-PCR was used to evaluate changes in the mRNA expression levels after reverse transcription of 1 μg of RNA from each sample using the PrimeScript RT reagent kit (Takara Bio, Inc., Otsu, Japan). SYBR Premix Ex Taq technique was used for qRT-PCR using a light cycler instrument (Roche Diagnostics, Mannheim, Germany) (Takara Bio). The B2m gene was used as a normalizer, and the rate of change in expression of the targeted genes was compared to that of the normalizer based on the 2-ΔΔCt relative expression formula. The genes and their primers are listed in Table 1.

**Table 1:** Primer sequences used in PCR amplification.

Primer	Forward sequence	Reverse sequence
B2m	GGAAGTTGGGCTTCCCATTCT	CGTGATCTTTCTGGTGCTTGTC
TNF-α	TAGCCATTGTGAAGGAGGGC	CCTGAGGCCGTTCCTTGTAG
IL−1β	GCTCCAGCACTATGTCACCA	CGTCTGAGCTGGAAACCAGT

### 2.7 Statistical analysis

The data were presented as Mean±SD. Normality of distributions was tested with the Shapiro-Wilk test. The homogeneity of variance was checked with Levene's test. The data were analyzed, and graphs were plotted using GraphPad Prism software version 9. T-test and One-way ANOVA followed by Tukey’s post-test were used for data analysis. The P<0.05 is considered a significant level.

## 3 Results

### 3.1 Seizure activity

**Fig. 1:**
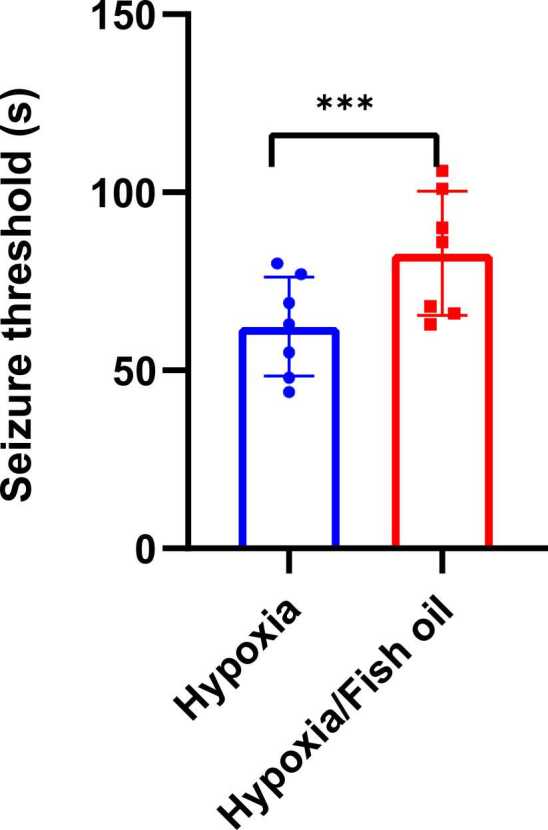
Comparison of the average duration required for the onset of the first convulsive activity (seizure threshold) in the hypoxia groups treated with fish oil or saline. The data were presented as Mean±SD and analyzed with t-test. *** indicates a significant difference between the hypoxia group treated with fish oil compared to the hypoxia group that received saline (p<0.001) (n=7).

The seizure threshold in the hypoxia group treated with fish oil increased significantly compared to the hypoxia group (p=0.0006) (Fig. 1).

**Fig. 2:**
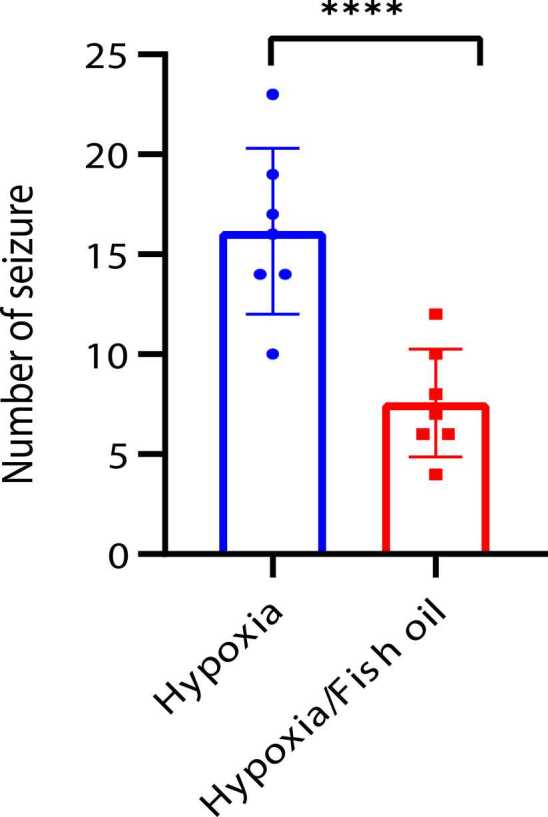
Comparison of the average number of seizures in the hypoxia groups treated with fish oil or saline. The data were presented as Mean±SD and analyzed with t-test. **** indicates a significant difference between the hypoxia group treated with fish oil compared to the saline-treated hypoxia group (p<0.0001) (n=7).

The number of tonic-clonic seizures in the hypoxia group treated with fish oil was significantly reduced compared to the saline-treated hypoxia groups (p=0.0001) (Fig. 2).

### 3.2 Open field test assay

**Fig. 3:**
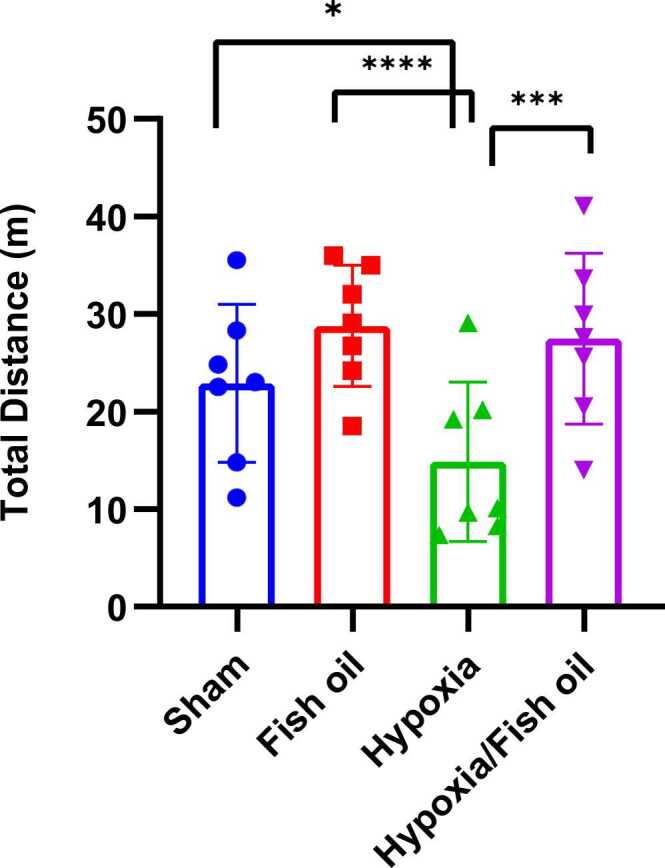
Comparison the average of traveled distance in the open field between the studied groups. The data were presented as Mean±SD and analyzed with one-way ANOVA followed by Tukey’s post-test.* indicates a significant difference between the hypoxia group compared to the control (sham) group (p<0.05) and **** indicates a significant difference between the hypoxia group and the fish oil group (p<0.0001). *** shows a significant difference between the oil fish/hypoxia group compared to the hypoxia group (p<0.001) (n=7).

In this test, the average distance traveled, movement speed,and the number or frequency of rearing were measured.The results showed that a significant decrease in the average total distance traveled in the open field in the hypoxia group compared to the control (sham) group (p=0.0185) and fish oil (p=0.0) groups; While the consumption of fish oil during lactation caused a significant increase in the total displacement in the hypoxia fish oil group compared to the hypoxia group alone (p=0.0002) (Fig. 3).

**Fig. 4:**
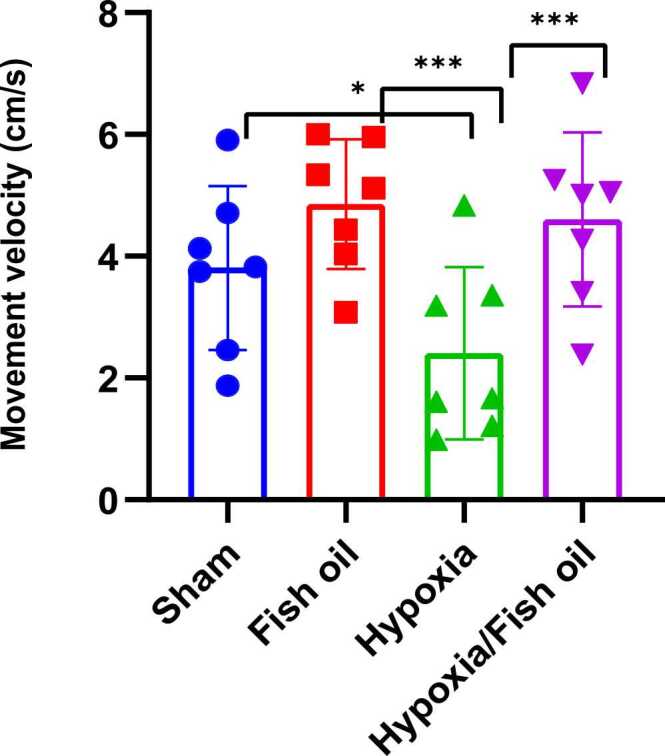
Comparison of the average movement speed (cm/s) between the studied groups. The data were presented as Mean±SD and analyzed with one-way ANOVA followed by Tukey’s post-test.* indicates a significant difference between the hypoxia group compared to the control (sham)group (p < 0.05) and *** indicates a significant difference between the hypoxia group compared to the fish oil and hypoxia fish oil groups (p < 0.001) (n=7).

The average speed of movement in the hypoxia group significantly decreased compared to the control (sham) (p=0.0409) and fish oil (p=0.0001) groups. Also, the consumption of fish oil during lactation caused a significant improvement in the speed of movement in the hypoxia fish oil group compared to the hypoxia group (p=0.0001) (Fig. 4).

**Fig. 5:**
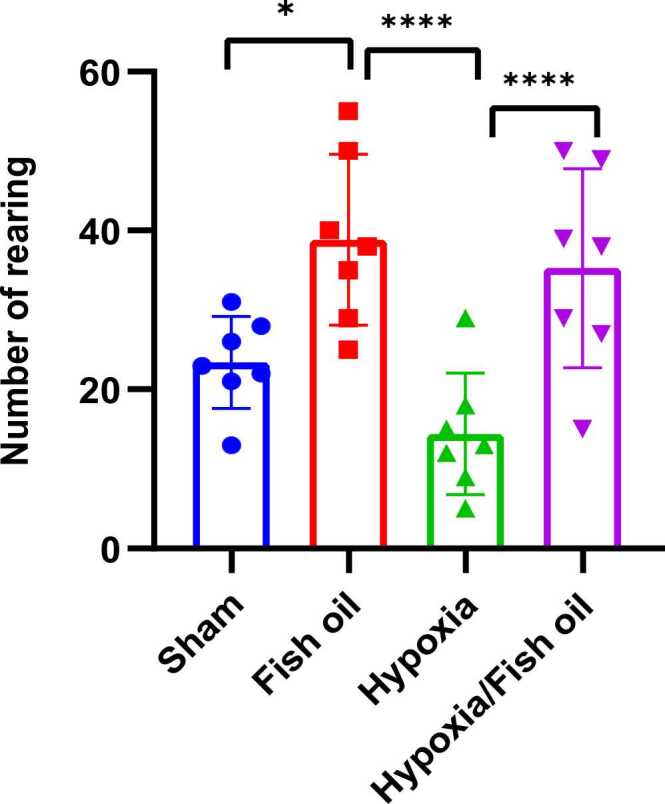
Comparison the average of rearing frequency between the studied groups. The data were presented as Mean±SD and analyzed with one-way ANOVA followed by Tukey’s post- test. * indicates a significant difference between the fish oil group compared to the control group (p<0.05) and **** indicates a significant difference between the hypoxia group and the fish oil and hypoxia-fish oil groups (p<0.0001) (n=7).

The results of the average frequency of standing on hind limbs (Rearing) show a significant increase in the group that received fish oil compared to the control (p=0.0167) and hypoxia (p=0.0001) groups. It was also found that the consumption of fish oil during breastfeeding caused a significant increase in rearing in the hypoxia-fish oil group compared to the hypoxia group alone (p=0.0001) (Fig. 5).

### 3.3 Rotarod assay

**Fig. 6:**
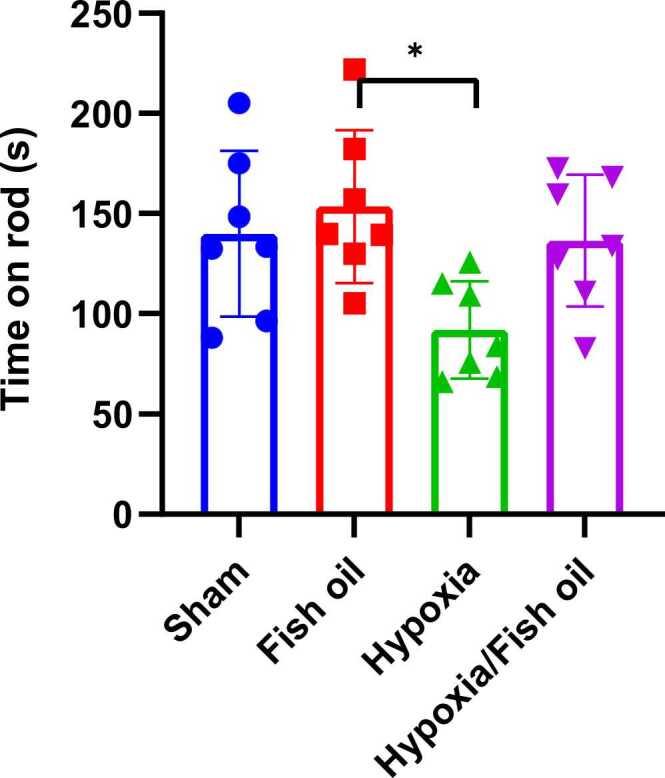
Comparison the average of time staying on the rotating rod (in seconds) between the studied groups. The data were presented as Mean±SD and analyzed with one-way ANOVA followed by Tukey’s post-test. * indicates a significant difference between the hypoxia group compared to the fish oil group (p<0.05) (n=7).

The obtained results show that the average duration of maintaining balance in the hypoxia group rats decreased significantly compared to the fish oil received group (p=0.0153). However, no significant difference was observed between the control group and the fish oil (p=0.8407), hypoxia (p=0.0784), and hypoxia fish oil (p=0.9977) groups (Fig. 6).

### 3.4 Wire hanging test

**Fig. 7:**
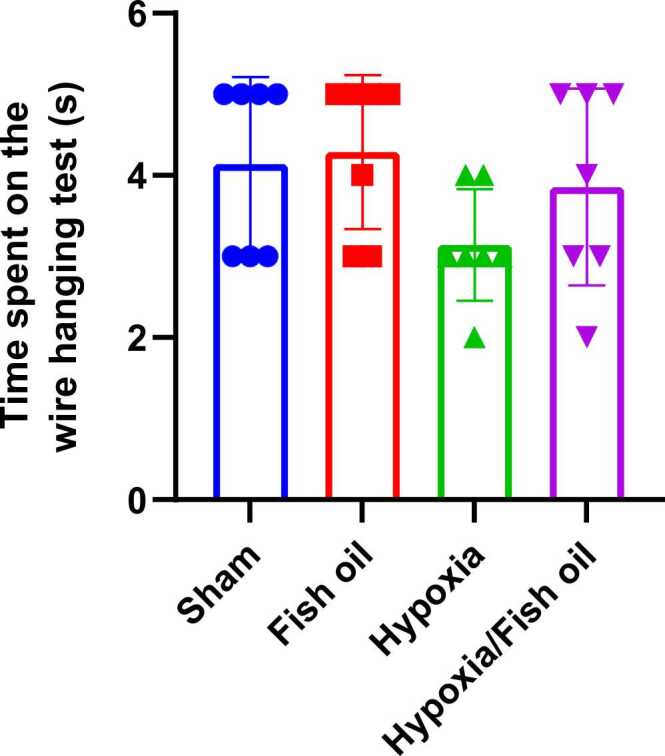
Comparison of the average score obtained in the reverse mesh test between the studied groups. The data were presented as Mean±SD and analyzed with one-way ANOVA followed by Tukey’s post-test. No significant differences were observed between the groups (n=7).

The statistical analysis of one-way analysis of variance showed that the average score obtained in the hypoxia group was lower than the other groups, but this difference was not statistically significant (Fig. 7).

### 3.5 Novel object test

**Fig. 8:**
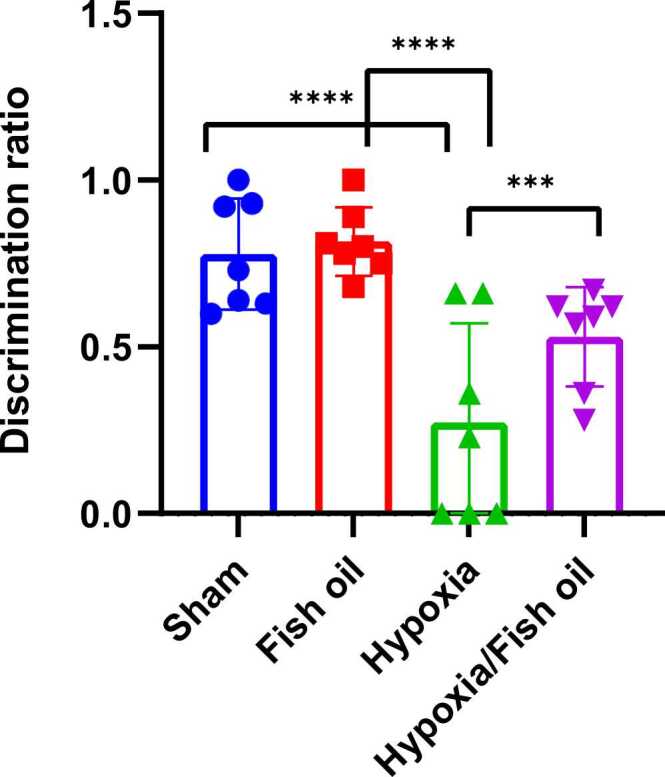
Comparison of average Discrimination ratio between the studied groups. The data were presented as Mean±SD and analyzed with one-way ANOVA followed by Tukey’s post- test. **** indicates a significant difference between the hypoxia group and the control and fish oil groups (p<0.0001) and *** indicates a significant difference between the hypoxia-fish oil group and the hypoxia group (p<0.001) (n=7).

The Discrimination ratio index showed that the ratio of the time spent around the new object to the total search time around both objects was lower than 0.5 in most of the rats in the hypoxia group, and the index was higher than 0.5 in all of the rats in the control group. Moreover, fish oil group and most of the rats in the hypoxia fish oil group were above 0.5 indicating a significant decrease in this ratio in the hypoxia group compared to the control (p < 0.0001), fish oil (p < 0.0001) and hypoxia fish oil (p = 0.0007) groups (Fig. 8).

**Fig. 9:**
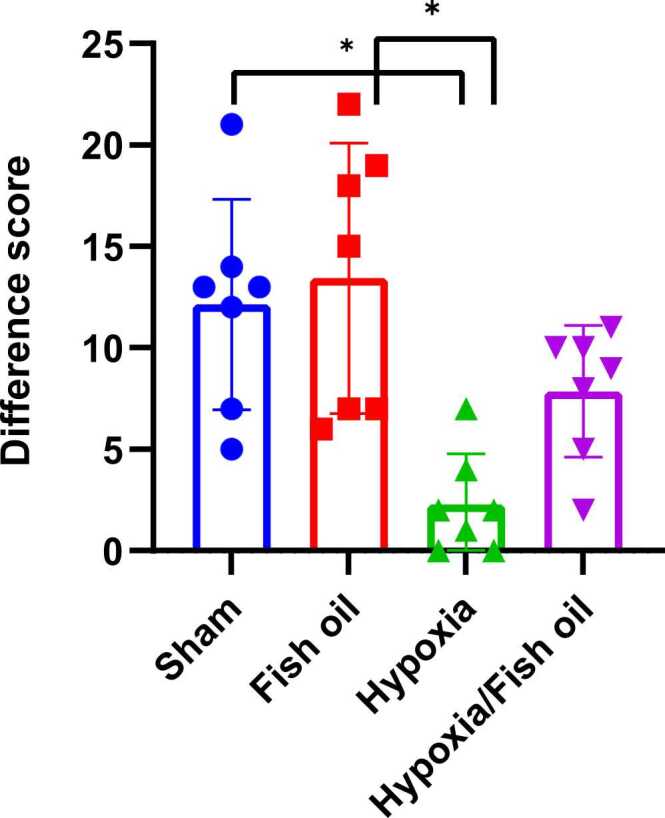
Comparison of the average Difference score (in seconds) between the studied groups. The data were presented as Mean±SD and analyzed with one-way ANOVA followed by Tukey’s post-test.* indicates a significant difference between the hypoxia group compared to the control and fish oil groups (p<0.05 (n=7).

It was also shown that the average difference score of most hypoxia group rats is below zero and closer to negative values; While in the control and fish oil groups, all the data were evaluated above zero and positive, and in the hypoxia fish oil group the time difference between the new and old object for most rats was above zero and positive. According to the obtained results, a significant difference was observed in this score between the hypoxia group, the control (p=0.0175) and the fish oil group (p=0.0127) (Fig. 9).

### 3.6 Histomorphometric examination of the hippocampus

**Fig. 10:**
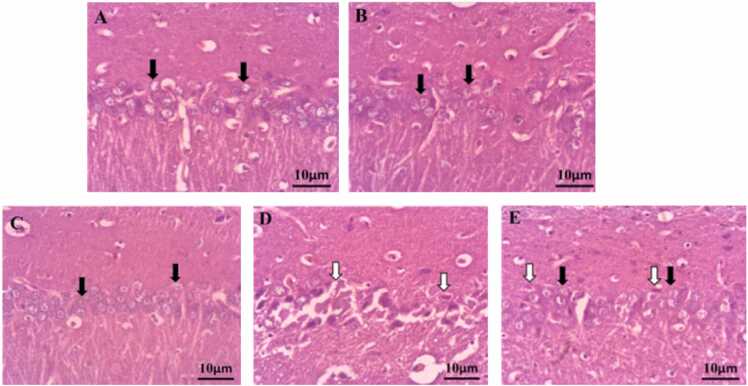
Micrograph of hippocampus CA1 molecular layer with H&E staining in groups A) Control, B) sham, C) fish oil, D) hypoxia,and E) hypoxia fish oil. Feathered arrow: clear cells with distinct round nuclei. Hollow arrow: wrinkled cells with necrotic nuclei.

In the microscopic observation of the brain, the CA1 molecular layer was checked, and in the sham and oil fish groups, the structure of the cells in this layer is completely normal, and the cell bodies of the neurons are small, healthy, and no significant difference was observed between these two groups (photos A, B, C in Fig. 10).

In the hypoxia group, a significant decrease in the number and arrangement of molecular layer cells was observed compared to the sham and oily fish groups (p<0.0001). In this group, a large number of cells in the CA1 molecular layer of the hippocampus have been destroyed, and their cytoplasm is dense, small and dark, which indicates the occurrence of necrosis in the cells (Photo D in Fig. 10). The number of cells in the CA1 molecular layer of hippocampus in the hypoxia fish oil group decreased compared to the sham group, but showed a significant increase compared to the hypoxia group (Table 2).

**Table 2:** The comparison of the Mean ± SD of diameter, the number of cells in the CA1 molecular layer, number, diameter and height of Purkinje cells, and the thickness of the molecular layer of the cerebellum in different groups. The letters a, b, and c indicate the significant difference between different groups (p<0.05).

Markers	Sham	Fish oil	Hypoxia	Hypoxia/Fish oil
Number of pyramidal cells of CA1 (N/mm)	^a^290.2±15.6	^a^340.5±28.4	^c^ 84.2±9.5	^b^168.6±12.4
Number of Purkinje cells (PC/mm)	^b^64.2±8.4	^a^72.0±8.6	^c^ 33.5±5.2	^b^50.4±2.1
Diameter of Purkinje cells (μm)	^a^19.0±2.5	^a^26.0±4.9	^c^12.0±2.8	^b^16.0±3.5
Height of Purkinje cells (μm)	^ab^9.7±0.5	^a^14.1± 0.9	^c^6.0±0.1	^b^8.6±0.9
Molecular layer thickness (μm)	^b^58.1±9.8	^a^64.2±8.9	^c^54.1±6.5	^b^56.3±7.4

### 3.7 Histomorphometric examination of the cerebellum

**Fig. 11:**
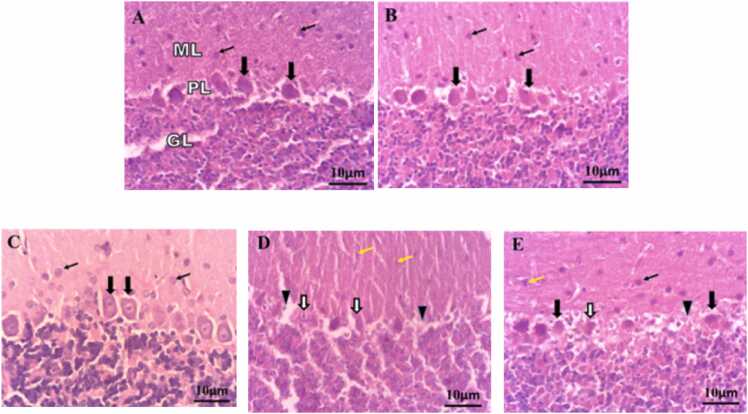
Micrograph of the molecular, Purkinje,and granular layers of the cerebellum with H&E staining. A) control, B) sham, C) fish oil, D) hypoxia,and E) hypoxia fish oil. The filled arrow indicates normal Purkinje cells and the hollow arrow indicates dense and damaged Purkinje cells, the arrowhead indicates the absence of Purkinje cells, the thin black arrow indicates healthy cell bodies in the molecular region, and the thin red arrow indicates condensed cell bodies in the molecular region.

In the microscopic observation of the cerebellum, all three molecular, Purkinje, and granular layers were examined and as seen in Fig. 11, these 3 layers in the sham and fish oil groups are normal and the cell bodies of the neurons are small, healthy, normal, and there is no a significant difference between these two groups (photos A, B, C in Fig. 11).

In the fish oil group, the number of Purkinje cells increased significantly compared to other groups (Table 2). But in the hypoxia group, Purkinje's layer cells have damage with microscopic changes. Following hypoxia, a large number of Purkinje cells have been destroyed, and in other cells, the lack of a nucleus, cell folds, and darkening of the cytoplasm can be clearly seen and recognized, and in this group, the number of cells in the Purkinje layer is significantly reduced compared to other groups.

Also, in the molecular layer of the cerebellum, the cell bodies of neurons were seen to be smaller and darker in staining (photo D in Fig. 11). It is noteworthy that pretreatment with fish oil had a protective role in different layers of the cerebellum and its constituent cells, and the lesions in the hypoxia fish oil group were significantly reduced compared to the hypoxia group (Photo E in Fig. 11).

In the histometric study of the cerebellum, the diameter and height of Purkinje cells and the thickness of the molecular layer of the cerebellum were also measured, and as seen in Table 1, the diameter and height of Purkinje cells in the hypoxia group were significantly reduced compared to other experimental groups, and pretreatment with fish oil increased the diameter and also the height in the hypoxia fish oil group were significantly increased compared to the hypoxia group (p<0.05). The results of measuring the thickness of the molecular layer showed that the thickness of this layer increased significantly in the fish oil group compared to other groups; While in the hypoxia group, it shows a significant decrease compared to other experimental groups (p<0.05). The increase in the thickness of the molecular layer in the hypoxia fish oil group compared to the hypoxia group is also significant (p<0.05) (Table 2).

### 3.8 Proinflammatory factors investigation

**Fig. 12:**
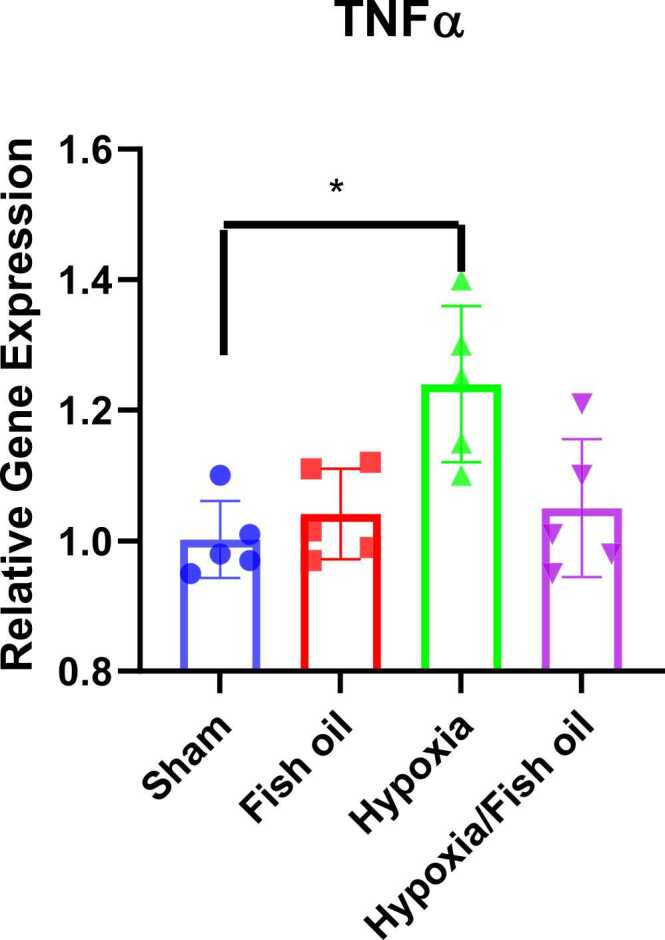
Comparison of the TNF-α gene expression between the groups. The data were presented as Mean±SD and analyzed with one-way ANOVA followed by Tukey’s post-test. * indicates a significant difference between the hypoxia group compared to the control group (p< 0.05) (n=5).

**Fig. 13:**
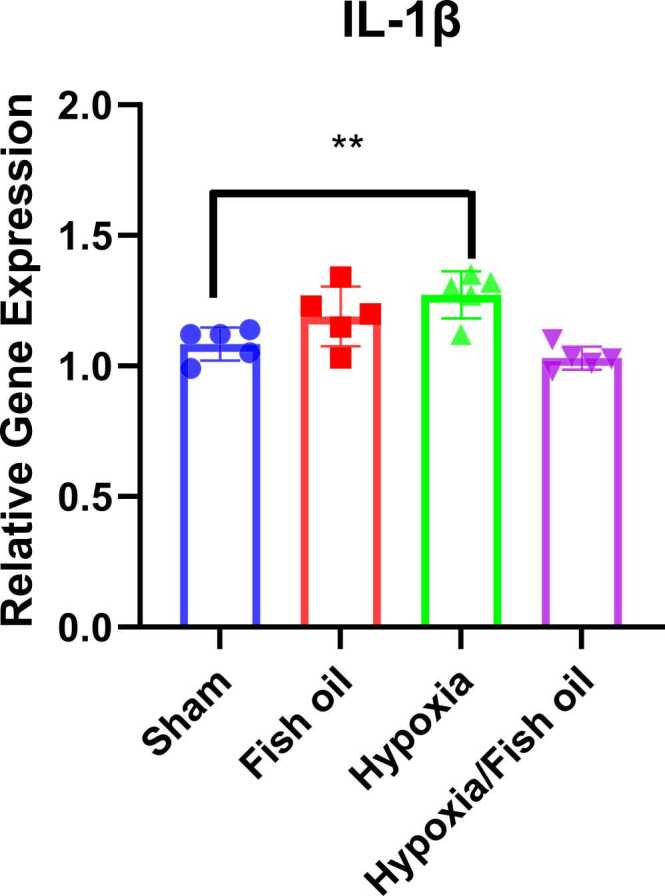
Comparison of the IL-1β gene expression between the groups. The data were presented as Mean±SD and analyzed with one-way ANOVA followed by Tukey’s post-test. ** indicates a significant difference between the hypoxia group compared to the control group (p<0.01) (n=5).

Our results demonstrated that in a rat model of hypoxia, the gene expression of pro-inflammatory cytokines, TNF-α (p<0.05) and IL-1β (p<0.01) were increased compared to the control group (Fig. 12, Fig. 13).

## 4 Discussion

The findings obtained from this study show that applying hypoxia in 12-day-old neonate rats increases the excitability of neurons and the occurrence of spontaneous seizures. In line with these results, various studies have shown that applying hypoxia can increase neuronal excitability and induce seizures (Abbasi and Unsworth, 2020). It has been determined that after a seizure attack, the contraction of blood vessels causes a further decrease in blood supply to brain areas and, as a result, a decrease in oxygen levels in these areas (Farrell et al., 2016). The mammalian brain strongly depends on an adequate and continuous supply of oxygen. Its necessity is due to the production of adenosine triphosphate (ATP), the primary source of cellular energy required to maintain the structural and functional integrity and maintain the proper functioning of the brain. Too little or too much oxygen in the blood can have serious consequences. This high sensitivity to changes in oxygen concentrations can make the central nervous system highly vulnerable to changes in oxygen concentration. Although neurons die during prolonged hypoxia, the primary cellular response before functional arrest and cell death is cell membrane depolarization. This initial hypoxic depolarization becomes increasingly important during brain pathological states such as epilepsy. Previous reports indicate that hypoxia is a source of cellular hyperexcitability and the main cause of some epileptic seizures (Ingram et al., 2014).

It seems that the mechanism of seizures in infants is different from that in adults or even in childhood. Most neonatal seizures occur as a transient response to acute brain pathology. In infants with HIE, seizures usually begin hours after birth, their severity reflects the severity of the underlying brain injury (Zhou et al., 2021). Biologically, there are differences in neurotransmitter functions during developmental periods that may increase the susceptibility of the immature brain to seizures, and have the potential to affect the efficacy of anticonvulsant drugs. Early in development, gamma-aminobutyric acid (GABA) receptors may become excitatory following stimulus rather than the inhibition seen in adults. This difference is due to the high concentration of intracellular chloride caused by the high expression of Na-K-2Cl (NKCC1), which mediates the entry of chloride, and the decrease in the expression of KCC2, which mediates the exit of chlorine from the cells. Following the upregulation of the potassium-chloride transporter KCC2 in the early postnatal period, Cl- can be extruded from the cells and thus GABA and glycine are inhibited. During the embryonic period, it has been shown that hypoxia can reduce the number of internal neurons or the number of synapses and thus affect the frequency of GABA currents. Altered delayed hyperpolarization of GABA action as a main mechanism causes exacerbation of excitability and as a result, causes disorders such as epilepsy and autism. This relative increase in excitability is important for the development of neural circuits, but is likely to increase susceptibility to seizures. Impaired GABAergic signaling after hypoxia may lower the seizure threshold and contribute to the reduced effect of GABAergic agonists such as phenobarbital in neonates (Nardou et al., 2013), 34, 35, 36, (Yakovlev et al., 2023).

We observed seizures attacks, increased inflammation, a significant increase in atrophy of cells causing tissue damage in the hippocampus and cerebellum, and impaired memory and locomotion following hypoxia. Various factors, including hypoxia, lead to neuroinflammation. Hypoxia is strongly associated with dementia. Chronic hypoxia stimulates neuroinflammation by activating microglia, and brain-resident immune cells, along with increased reactive oxygen species and pro-inflammatory cytokines which are associated with many degenerative disorders of the central nervous system (CNS) (Hambali et al., 2021). Chronic hypoxia affects cellular metabolism, ATP production, calcium homeostasis, ROS production, and inflammation. In addition to chronic hypoxia, acute hypoxia also causes the production of pro-inflammatory cytokines and chemokines, including IL-6, and TNF-α. Short-term and acute hypoxia also increased the expression of TNF-α, IL-1β, and oxidative stress-related genes. Multiple mechanisms are involved in the pathogenesis of neonatal hypoxic brain injury, including oxidative stress, apoptosis, inflammatory reactions, and excitotoxicity (Zhang et al., 2017, Wu et al., 2015).

The inflammatory cascade is characterized by the rapid activation of resident microglia cells and the infiltration of peripheral leukocytes into the damaged parenchyma. Astroglia cells are also activated and play an important and stable role in inflammation following trauma and hypoxia (Kaur et al., 2013). Once activated, these inflammatory cells contribute to neuronal injury by releasing cytotoxic mediators, including cytokines, chemokines, reactive oxygen species, adhesion molecules, and matrix metalloproteinase (Stigger et al., 2013).

In the study, the expression of pro-inflammatory genes increases in the first 2 hours after hypoxia-ischemia in the neonatal brain. The expression levels of TNF-*α* and IL-1*β* were significant even 6 hours after the hypoxic attack. Inflammatory events were associated with the activation of quiescent glial cells. Twenty-four hours after the hypoxia, extensive activation and proliferation of microglial and astroglia cells were observed in the cortex and hippocampus. Activation of glial cells increases the inflammatory process and aggravates brain damage (Chen et al., 2015). Research has shown a positive correlation between the expression levels of inflammatory cytokines and chemokines following hypoxia-induced brain damage in infants (Orrock et al., 2016).

Therapeutic approaches targeting and activating the anti-inflammatory pathway can reduce brain damage in animal models of neonatal hypoxia. Fish oil has been shown to exhibit neuroprotective effects in various models of CNS diseases. The results of our research showed that treating hypoxic rats with fish oil during lactation significantly increased the seizure threshold and decreased the number of tonic-clonic seizures. Also, pre-treatment with fish oil reduced inflammation and improved behavioral indicators (memory and movement) compared to the hypoxia group. Fish oil can cross the mother's milk in rats through different mechanisms, such as passive diffusion, active transport, or lipoprotein-mediated transport. The rate and extent of fish oil transfer depend on several factors, such as the concentration of fish oil in the mother's diet, the composition of the milk fat globules, and the presence of other lipids or proteins in the milk. Fish oil supplementation through milk can affect the cognitive abilities and neural development of rats (Faccinetto-Beltrán et al., 2022).

Fish oil containing Docosahexaenoic (DHA) and Arachidonic acid, which are essential fatty acids, prevent lipid peroxidation and protect tissue against oxygen free radicals. The amount of DHA in breast milk directly depends on the mother's diet. Consuming a DHA-rich diet leads to a dose-dependent increase in the amount of DHA in breast milk. Normally, the amount of DHA in the tissues of breastfed infants is high. Studies show that n-3 fatty acids reduce the excitability of neurons and modulate the activity of sodium and calcium channels (Serrano et al., 2023, DeGiorgio and Taha, 2016).

Fatty acids affect inflammation through different mechanisms. Many of these affect inflammation by changing the fatty acid composition of the cell membrane. Following these changes, membrane fluidity and cell signaling also change. Eicosanoids produced from arachidonic acid have anti-inflammatory effects. PUFA with anti-inflammatory properties has a therapeutic effect in rheumatoid arthritis. The anti-inflammatory effects of marine n-3 PUFAs may also contribute to their protective effect against atherosclerosis, plaque rupture, and cardiovascular mortality (Calder, 2010, Panezai and van Dyke, 2023).

It has been found that DHA has extensive anti-inflammatory effects by activating Nrf2-dependent antioxidant responses and inhibiting cytokine production and nitric oxide expression in activated macrophages (Groeger et al., 2010). DHA plays a role in the synaptogenesis of neurons, brain growth, synaptic plasticity and activity, and maintenance of neuronal membranes. It also participates in phosphatidylserine- dependent signaling pathways and it causes the differentiation of stem cells into neurons (Yassine et al., 2023, Kim and Spector, 2018). Many studies pointed to an increase in DHA, DPA and a reduction in the risk of cognitive disorders (Samieri et al., 2012).

Dietary enrichment of aged rats with EPA, and DHA has a positive effect on LTP-related impairments in the aged, and these effects are likely mediated through multiple anti-inflammatory effects acting through changes in cytokine levels. For example, 8 weeks of dietary supplementation with 10 mg/day of DHA inhibits age-related impairments of LTP and depolarization-induced glutamate release. Similarly, feeding mice with an EPA-containing diet for 8 weeks (10 mg/day for 3 weeks and 20 mg/day for 5 weeks) prevented the increase in IL-1β in the cerebral cortex and hippocampus and also restored LTP (Martin et al., 2002).

Brain histomorphometric studies in this study showed that hypoxia significantly causes the destruction and necrosis of cells of the molecular layer CA1 of the hippocampus, which was significantly controlled by treatment with fish oil. It was also observed in the cerebellum that the application of hypoxia causes a significant reduction of Purkinje cells and other mentioned tissue damage, and these cases were significantly modified by the consumption of fish oil. It is worth noting that following administration of oil fish, the number of Purkinje cells increased significantly. In this regard, it was previously shown that pyramidal cells in the CA1 region are more sensitive to ischemia-hypoxia than other regions of the hippocampus (Nakajima et al., 2015). Following ischemia-hypoxia, the structure of the hippocampus is disrupted,and the cells are arranged irregularly, and cellular edema and necrosis of nerve cells are observed in the brain tissue (Jiang et al., 2020). It has also been determined that in the cerebellum, Purkinje cells have the highest sensitivity to hypoxia, which is most likely due to their high cellular metabolism and their high dependence on oxygen (Hausmann et al., 2007, Witter et al., 2016). Cerebellar injury reduces the number of Purkinje cells and changes the morphology and activity of cerebellar tissue. These changes include increased activity of microglial cells along with increased pro-inflammatory cytokines, and decreased size and density of Purkinje cells (Zhang et al., 2019). It has been reported that the application of hypoxia causes damage in Purkinje cells (Leroux et al., 2022).

## 5 Conclusion

The results obtained from this research showed that applying hypoxia in neonatal rats, which is a sensitive period of synaptic plasticity, increases the number of tonic-clonic seizures. Pro-inflammatory cytokines increase in adult rats. A decrease in the number of neurons in the areas that control locomotion, and the formation of long-term memory and behavioral disorders, has been seen in the hypoxia rats. Pretreatment with fish oil during lactation, protects neurons from inflammation caused by hypoxia, leads to a decrease in irritability, an increase in the seizure threshold, and reduces neuronal death and brain histomorphometric changes, it also prevents memory and movement disorders in the long term.

## Ethics approval (research involving animals)

Animal studies were conducted according to the protocols and guidelines approved by the Institutional Ethics Committee of Shahid Chamran University of Ahvaz (EE/98.24.3.26545/Scu.ac.ir) and were conducted under the Guide for the Care and Use of Laboratory Animals (NIH).

## Funding

We are grateful to the Research Council of the Shahid Chamran University of Ahvaz for financial support.

## CRediT authorship contribution statement

**Zahra Basir:** Writing – original draft, Methodology. **Hossein Amini Khoei:** Writing – original draft, Software, Methodology. **Nima Badripour:** Writing – original draft, Project administration, Methodology. **Shima Balali Dehkordi:** Data curation, Investigation, Methodology. **Zohreh Ghotbeddin:** Writing – original draft, Supervision, Data curation, Conceptualization.

## Declaration of Competing Interest

The authors have no conflicts of interest to declare regarding the study described in this article and preparation of the article.

## Data Availability

The datasets generated for this study are available on request to the corresponding author.
